# Clinical and Multimodal Imaging Features of Subretinal Drusenoid Deposits

**DOI:** 10.18502/jovr.v16i2.9082

**Published:** 2021-04-29

**Authors:** Devesh Kumawat, Srikanta K. Padhy, Vinod Kumar

**Affiliations:** Dr Rajendra Prasad Centre for Ophthalmic Sciences, All India Institute of Medical Sciences, New Delhi, India

**Keywords:** Subretinal Drusenoid Deposits, Pseudodrusen, Multimodal Imaging, Optical Coherence Tomography, Age-related Macular Degeneration

## Abstract

**Purpose:**

To describe the multimodal imaging (MMI) features of subretinal drusenoid deposits (SDD) in Indian population.

**Methods:**

Patients diagnosed to have SDD from January 2016 to December 2018 at our tertiary care center were recruited. The diagnosis of SDD was made on the basis of MMI consisting of a combination of color fundus photography (CFP), optical coherence tomography (OCT), red-free (RF) imaging, blue autofluorescence (BAF), and near-infra red reflectance (NIR) imaging. The morphological type and distribution of SDD and the associated retinal lesions were reviewed.

**Results:**

Twenty-three patients with SDD were included. The mean age of the patients was 68.1 ± 12.2 years. SDD were noted in 77.8% of eyes clinically (*n* = 35/45) and could be detected in 100% of these eyes with OCT. The morphology of SDD was nodular in 65.7% of eyes (*n* = 23/35), reticular in 5.7% (*n* = 2/35), and mixed pattern in the remaining cases. BAF and NIR showed hyporeflective nodular lesions often with a target configuration. The location was commonly in the perifoveal area, mostly involving the superotemporal quadrant (74.3%, *n* = 26/35). Associated retinal lesions were type-3 neovascularization or retinal angiomatous proliferation in 17.1% (*n* = 6/35), disciform scar in 11.4% (*n* = 4/35), type-1 neovascularization in 8.5% (*n* = 3/35), and geographic atrophy in 5.7% (*n* = 2/35) of eyes. The mean subfoveal choroidal thickness was 186.2 ± 57.8 µm.

**Conclusion:**

SDD commonly have a nodular morphology and their identification often requires confirmations with OCT. Advanced age-related macular degeneration features are frequently present in eyes with SDD and the fellow eyes.

##  INTRODUCTION

Drusen associated with pigmentary changes are risk factors for late age-related macular degeneration (AMD) and related ocular morbidity.^[1,2,3]^ Characteristically, drusen are extracellular deposits between the basal lamina of retinal pigment epithelium (RPE) and the inner collagenous layer of the Bruch's membrane. Subretinal drusenoid deposits (SDD) also known as reticular pseudodrusen (RPD) are a distinct entity.^[4,5,6]^ These deposits occur in the subretinal space above the RPE and are seen predominantly in the perifoveal area. These may also be seen in the mid-periphery of the retina. Initial description of SDDs was using blue filters in fundus imaging; however, it was difficult to distinguish them clinically. With the arrival of modern high-resolution optical coherence tomography (OCT), these can be picked up with high sensitivity and specificity.^[7,8]^ Additional imaging investigations such as near infra-red reflectance (NIR) imaging, red-free fundus (RF) imaging, and short-wave or blue autofluorescence (BAF) may help increase the detection rates.^[7]^ SDD have been found to be independent risk factors for late AMD.^[9,10]^ A strong association has been noted between these and type-3 intraretinal neovascularization,^[11,12]^ outer retinal atrophy,^[13,14]^ and combined outer retinal and RPE atrophy or geographic atrophy.^[9,10][15]^

Although SDD have been studied in detail in the past few years, there are no studies describing SDD in the Indian population. This study aims to describe the demography, clinical characteristics, and multimodal imaging (MMI) features of SDD in the Indian population.

##  METHODS

This retrospective observational study was performed at a tertiary eye care center in North India. The study adheres to the tenets of the Declarations of Helsinki and to the institutional guidelines for research. Informed consent was obtained from all the patients. The participants were the cases of SDD diagnosed at our center over a period of three years (2016 to 2018). The clinical database from the retina lab was reviewed to identify the patients.

**Figure 1:**
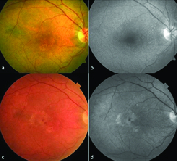
Multimodal imaging of a case of SDD. (a) Fundus photograph shows multiple dot-like regularly arranged array of whitish deposits predominantly in the perifoveal area. (b) The lesions are better delineated on blue channel or red-free photograph. (c) Fundus photograph after a year shows presence of intraretinal hemorrhage and neovascular membrane suggesting the development of retinal angiomatous proliferation. The number of SDD has decreased. (d) The red-free photograph reveals only a few SDD in the nasal macula, suggesting the regression of SDD.

**Figure 2:**
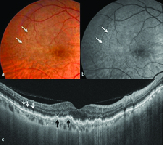
Multimodal imaging of a case of AMD. (a) Fundus photograph shows multiple soft drusen at the fovea with pigmentary changes suggestive of non-exudative AMD. Numerous confluent ribbon-like interlacing reticular drusen are seen predominantly in the perifoveal area in superotemporal quadrant (white arrows). (b) The reticular lesions (white arrows) are better delineated on red-free imaging. (c) The swept-source OCT through the top white arrow in subfigure “a” shows broad mound-shaped deposits in the subretinal space (white arrows) deflecting the ellipsoid zone inward suggestive of reticular drusen. The black arrows point to the sub-RPE deposits suggestive of soft drusen. The subfoveal choroid seems markedly thin with obliteration of the choriocapillaris layer.

**Figure 3:**
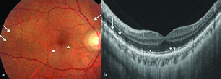
A case of mixed SDD. Fundus photograph shows dot-like SDD (white arrowheads) near the fovea and reticular lesions (white arrows) in the temporal perifoveal and peri-papillary area. (b) SS-OCT image through the white right arrow in subfigure “a” shows sharp peaked (white arrowheads) and broad mound-shaped deposits (white arrows) in the subretinal space suggestive of dot and reticular drusen, respectively.

**Figure 4:**
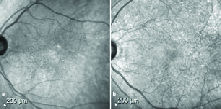
Near infra-red reflectance imaging of SDD. (a) Numerous clearly visible perifoveal SDD appearing as hyporeflective dots. (b) Numerous dot-like SDDs with a “target” configuration. The central core appears hyperreflective with a hyporeflective annulus.

The diagnosis of SDD was made on the basis of MMI consisting of a combination of either color fundus photography (CFP), RF imaging, BAF, NIR imaging, and OCT. The SDD appear as yellowish-white deposits on CFP, present more superficial than the conventional drusen. The size is similar to that of the hard drusen. These are of three clinical types: nodular or dot type with dot-like uniformly arranged lesions [Figure 1], reticular type with broad ribbon-like interlacing deposits [Figure 2], and mixed type with a combination of these two [Figure 3]. On OCT, these deposits appear as hyperreflective conical or smooth mounds [Figures 2 and 3], present in the subretinal space, and may erode into the ellipsoid zone as their stage progresses. Later, these may undergo resolution with loss of the outer retinal bands and RPE-Bruch's complex. RF or blue-channel imaging highlights these lesions better as compared to CFP [Figures 1 and 2]. On BAF, these lesions are often hypoautofluorescent with a hyperautofluorescent core sometimes seen in nodular types with increasing stage. NIR is also useful in picking up the nodular type of lesions and these appear as hyporeflective dots as well [Figure 4]. They may often have a “target” configuration with a central hyper reflective core and surrounding hyporeflective annulus.

**Figure 5:**
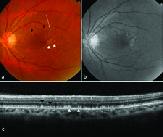
Multimodal imaging of a case of AMD. (a) Fundus photograph shows multiple soft drusen at the fovea (white arrowheads). Numerous dot-like SDD are seen predominantly in the perifoveal area (black arrowheads). (b) The SDD are better delineated than soft drusen on red-free imaging. (c) SD-OCT image through the white arrow in subfigure “a” shows sharp peaked subretinal dot-like SDD (black arrowheads) disrupting the ellipsoid zone and smooth mounds sub-RPE suggestive of soft drusen (white arrowheads).

**Figure 6:**
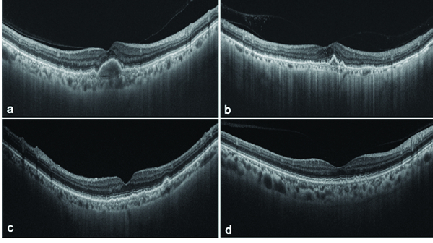
Swept source-based choroidal imaging in eyes with SDD (a–d). While “a” and “d” show near-normal choroidal morphology, the choroid is severely thinned out in “b” and “c”. The subfoveal choroidal thickness was 166, 74, 115, and 244 in “a”, “b”, “c”, and “d”, respectively.

The MMI imaging was performed using a swept-source platform (DRI Triton, Topcon Inc., Oakland, New Jersey, USA). In a subset of patients, the confocal scanning laser ophthalmoscope-based imaging system was used (Spectralis HRA, Heidelberg, Germany). Fundus fluorescein angiography (FFA) and indocyanine green angiography (ICGA) were performed in a subset of patients to identify/document the coexisting intraretinal/subretinal/sub-RPE neovascular complex.

The details such as demography, visual acuity, clinical fundus features, and MMI characteristics were noted for each case. The key parameters noted were the morphological type and distribution of SDD, and the associated retinal lesions.

Data were recorded on an Excel spreadsheet and analyzed with STAT 12.1 software. The parametric variables were represented with mean and SD, while the nonparametric variables were represented with median and range. The visual acuity was converted into the logMAR scale with counting fingers (CF) vision at 1 feet distance corresponding to 2.3 units.

##  RESULTS

Twenty-three patients with SDD were included. One eye of a patient had phthisis bulbi, therefore, a total of 45 eyes were finally studied. The mean age of the patients was 68.1 ± 12.2 years. Of the 23 patients, 19 (78.3%) were >65 years old. The majority of patients were females (*n* = 16/23, 69.6%) with a male-to-female ratio of 0.43:1.

SDD were clinically noted in 35 (77.8%) eyes. The SDD could be detected in all these eyes with OCT. Bilateral involvement was seen in 52.2% of the cases (*n* = 12/23). Morphology of SDD was nodular in 23 eyes (65.7%), reticular in 2 eyes (5.7%), and mixed pattern in the remaining 10 eyes (28.6%). The spatial location was commonly in the perifoveal area, mostly involving the superotemporal quadrant (74.3%, *n* = 26/35) [Table 1] followed by annular distribution (22.5%, *n* = 8/35). On studying the spatial location separately for different morphological types of SDD (see Table 1), the superotemporal quadrant was involved in 19 of 23 eyes (82.6%) and 6 of 10 eyes (60%) with nodular and mixed types, respectively.

**Table 1:** Spatial distribution of subretinal drusenoid deposits or pseudodrusen


Location of SDD${}^{*}$	**Number of eyes (%)**			
	**Total (** ***n*** ** = 35)**	**Nodular type (** ***n*** ** = 23)**	**Reticular type (** ***n*** ** = 2)**	**Mixed type (** ***n*** ** = 10)**
Superotemporal quadrant	26 (74.3)	19 (82.6)	1 (50)	6 (60)
Inferotemporal quadrant	7 (20)	5 (21.7)	0	2 (20)
Superonasal quadrant	6 (17.1)	5 (21.7)	1 (50)	0
Inferonasal quadrant	2 (5.7)	2 (8.7)	0	0
Annular	8 (22.8)	3 (13)	1 (50)	4 (40)
${}^{*}$SDD, subretinal drusenoid deposits				

**Table 2:** Retinal features in eyes with subretinal drusenoid deposits


**Parameters**	**Number of eyes (%)**				
	SDD${}^{*}$ eyes				**Fellow eyes (** ***n*** ** = 10)**
	**Total (** ***n*** ** = 35)**	**Nodular type (** ***n*** ** = 23)**	**Reticular type (** ***n*** ** = 2)**	**Mixed type (** ***n*** ** = 10)**	
Soft drusen	24 (68.6)	16 (69.6)	1 (50)	7 (70)	2 (20)
Drusenoid PED${}^{†}$	3 (8.6)	1 (4.3)	–	2 (20)	–
Type 1 NV${}^{‡}$	3 (8.6)	1 (4.3)	–	2 (20)	–
Type 2 NV	–	–	–	–	1 (10)
Type 3 NV or RAP${}^{§}$	6 ((17.1)	3 (13)	1 (50)	2 (20)	2 (20)
Geographic atrophy	2 (5.7)	2 (8.7)	–	–	–
Disciform scar	4 (11.4)	4 (17.4)	–	–	7 (70)
${}^{*}$SDD, subretinal drusenoid deposits; ${}^{†}$PED, pigment epithelium detachment; ${}^{‡}$NV, neovascularisation; ${}^{§}$RAP, retinal angiomatous proliferation					

The average logMAR CDVA in eyes with SDD was 0.53 ± 0.65 (median 0.3, range 0 to 2.3). CDVA ≥ 20/40 was seen in 20 of 35 eyes (57.1%). Soft drusen [Figure 5] were present concurrently in 68.6% of eyes with SDD (*n* = 24/35), out of which three eyes had drusenoid PED as well. Associated retinal lesions in eyes with SDD were as follows (see Table 2): type-3 NV or retinal angiomatous proliferation (RAP) [Figure 1] in 17.1% (*n* = 6/35), disciform scar in 11.4% (*n* = 4/35), type-1 or sub-RPE CNV in 8.5% (*n* = 3/35), and geographic atrophy (GA) in 5.7% (*n* = 2/35) of the eyes. The mean subfoveal choroidal thickness (SFCT) was 186.2 ± 57.8 µm [Figure 6]. The SFCT was <125 µm (cut-off for age-related choroidal atrophy) in 17.1% (*n* = 6/35) and >250 µm in 14.3% of eyes (*n* = 5/35).

The fellow eyes without SDD (*n* = 10) had average logMAR CDVA of 1.8 ± 0.76 (median 2.3, range 0.6 to 2.3). Soft drusen were noted in 20% of these eyes (*n* = 2/10). Disciform scar, RAP, and type-2 or subretinal CNV were noted in 70% (7/10), 20% (2/10), and 10% (1/10) of the fellow eyes without SDD, respectively. The mean SFCT was 178.1 ± 50.4 µm in these eyes (obtained for nine eyes).

##  DISCUSSION

Recognition of SDD/RPD in AMD patients is important because it independently confers increased risk (above the usual risk associated with large soft drusen and pigmentary changes) of developing the late stage of AMD.^[9,10]^ In this study, SDD were associated with late AMD in 42.8% (15/35) of eyes. The risk of late AMD also increases in the fellow eyes if SDD are present.^[16]^

The RF or blue channel of CFP shows high specificity for SDD.^[7]^ The RPE normally filters off the blue light and therefore the soft drusen, which are present external to RPE, appear yellow. SDD lie internal to RPE and are easily visualized in blue light. Both the nodular and reticular types were visible better in blue channel CFP in the current study. The overlying SDD scatters the excitation light reaching the RPE and therefore SDD appear hypoautofluorescent on autofluorescence imaging.^[7]^

The patients in our study were mostly elderly (65+ years) females. Likewise, the population-based studies have also shown that SDD/PD are associated with increasing age and female gender, the odds ratio reported by the Rotterdam study being 1.2 and 2.1, respectively^[17]^ and that reported by Blue Mountains eye study being 3.4 and 2.0, respectively.^[18]^ In correlation with the existing literature,^[19]^ the nodular or dot type was the most common type in our patients. The reticular or ribbon type uncommonly occurs in isolation and only two eyes in our series had this morphological type. The majority of the SDD were located in superotemporal quadrant in our study. SDD start developing in the superior quadrants and later spread to the inferior hemi-retina.^[4]^ Previous studies have also shown that SDD occur preferentially in the perifoveal area corresponding to an annular area with high rod photoreceptor density.^[20]^ The impaired dark adaptation in eyes with SDD supports the hypothesis of origin of SDD from degenerating rod cells.^[21]^

Choroid is often severely thinned in eyes with SDD.^[8,22,23]^ In a series of 58 eyes with SDD, Zweifel et al reported severe choroidal thinning or choroidal atrophy (<125 µm) in 32 (55.2%) eyes.^[8]^ Although our study supports the observation of choroidal thinning in SDD, choroidal atrophy was noted in only 17.1% of eyes. Binarization studies have shown decreased choroidal vascularity in eyes with SDD.^[24,25]^ Since a few eyes with SDD also have normal or increased choroidal thickness (five eyes in this study), it is possible that the SDD are not directly responsible for the choroidal thinning. Instead, RPE dysfunction may be responsible for both SDD formation and choroidal atrophy.^[4]^

SDD are dynamic structures. With increasing stage, they reabsorb and fade out.^[8,14]^ The fellow eyes of 10 patients in our series did not have SDD but had advanced AMD characteristics like disciform scar and RAP lesions. Perhaps, the SDD had regressed and were therefore not picked up in these eyes.

Many studies have shown that late AMD commonly occurs in eyes with SDD.^[9,10][13,14][15][26]^ In a large series of 155 eyes with SDD, Cohen et al reported features of AMD including soft drusen in 65% (*n* = 101), CNV (active/ scarred) in 39.3% (*n* = 61), and GA in 17.4% (*n* = 27).^[26]^ In our series also, the rates of soft drusen, CNV, and GA (68.6%, 37.1%, and 5.7%, respectively) were similar to those reported by Cohen et al.^[26]^ Not only eyes with SDD had associated AMD, the fellow eyes also had choroidal atrophy and CNV in our series. In addition to the macular neovascular disease and GA, Spaide et al have also described outer retinal atrophy as a consequence of SDD.^[13]^

There are few limitations of the present study including its retrospective nature. As SDD can be missed on routine examination, the rate of clinical SDD may be higher if specifically searched for. The sample size is relatively small and the study is based on a tertiary eye center, which could have led to selection bias. The prevalence and morphological type of SDD in AMD cases was not noted, which might improve the understanding behind the association between SDD and AMD.

To conclude, the patients with SDD in Indian settings are usually elderly females, with nodular type being the most common and mostly present in the perifoveal superior quadrants of the fundus. Their identification often requires OCT in addition to the CFP. These are frequently associated with advanced AMD features in the same eye as well as in the fellow eye.

##  Financial Support and Sponsorship

Nil.

##  Conflicts of Interest

There are no conflicts of interest.

## References

[1] Bird AC, Bressler NM, Bressler SB, Chisholm IH, Coscas G, Davis MD, et al. An international classification and grading system for age-related maculopathy and age-related macular degeneration. The International ARM Epidemiological Study Group. *Surv Ophthalmol* 1995;39:367–374. 10.1016/s0039-6257(05)80092-x 7604360

[2] Bressler SB, Maguire MG, Bressler NM, Fine SL. Relationship of drusen and abnormalities of the retinal pigment epithelium to the prognosis of neovascular macular degeneration. The Macular Photocoagulation Study Group. *Arch Ophthalmol *1990;108:1442–1447. 10.1001/archopht.1990.01070120090035 1699513

[3] Klein ML, Ferris FL, Armstrong J, Hwang TS, Chew EY, Bressler SB, et al. Retinal precursors and the development of geographic atrophy in age-related macular degeneration. *Ophthalmology* 2008;115:1026–1031. 10.1016/j.ophtha.2007.08.030 17981333

[4] Spaide RF, Ooto S, Curcio CA. Subretinal drusenoid deposits AKA pseudodrusen. *Surv Ophthalmol* 2018;63:782–815. 10.1016/j.survophthal.2018.05.005 29859199

[5] Sivaprasad S, Bird A, Nitiahpapand R, Nicholson L, Hykin P, Chatziralli I, et al. Perspectives on reticular pseudodrusen in age-related macular degeneration. *Surv Ophthalmol* 2016;61:521–537. 10.1016/j.survophthal.2016.02.005 26994868

[6] Rabiolo A, Sacconi R, Cicinelli MV, et al. Spotlight on reticular pseudodrusen. *Clin Ophthalmol Auckl NZ* 2017;11:1707–1718. 10.2147/OPTH.S130165 PMC5614782 29033536

[7] Ueda-Arakawa N, Ooto S, Tsujikawa A, Querques L, Bandello F, Querques G. Sensitivity and specificity of detecting reticular pseudodrusen in multimodal imaging in Japanese patients. *Retina *2013;33:490–497. 10.1097/IAE.0b013e318276e0ae 23403515

[8] Zweifel SA, Spaide RF, Curcio CA, Malek G, Imamura Y. Reticular pseudodrusen are subretinal drusenoid deposits. *Ophthalmology* 2010;117:303–312.e1. 10.1016/j.ophtha.2009.07.014 19815280

[9] Schmitz-Valckenberg S, Alten F, Steinberg JS, et al. Reticular drusen associated with geographic atrophy in age-related macular degeneration. *Invest Ophthalmol Vis Sci* 2011;52:5009–5015. 10.1167/iovs.11-7235 21498612

[10] Finger RP, Wu Z, Luu CD, Jaffe GJ, Fleckenstein M, Mukesh BN, et al. Reticular pseudodrusen: a risk factor for geographic atrophy in fellow eyes of individuals with unilateral choroidal neovascularization. *Ophthalmology* 2014;121:1252–1256. 10.1016/j.ophtha.2013.12.034 PMC4047161 24518615

[11] Sawa M, Ueno C, Gomi F, Nishida K. Incidence and characteristics of neovascularization in fellow eyes of Japanese patients with unilateral retinal angiomatous proliferation. *Retina *2014;34:761–767. 10.1097/01.iae.0000434566.57189.37 24100709

[12] Chang YS, Kim JH, Yoo SJ, Lew YJ, Kim J. Fellow-eye neovascularization in unilateral retinal angiomatous proliferation in a Korean population. *Acta Ophthalmol* 2016;94:e49–e53. 10.1111/aos.12748 25981599

[13] Spaide RF. Outer retinal atrophy after regression of subretinal drusenoid deposits as a newly recognized form of late age-related macular degeneration. *Retina* 2013;33:1800–1808. 10.1097/IAE.0b013e31829c3765 23764969

[14] Querques G, Canouï-Poitrine F, Coscas F, Massamba N, Querques L, Mimoun G, et al. Analysis of progression of reticular pseudodrusen by spectral domain-optical coherence tomography. *Invest Ophthalmol Vis Sci* 2012;53:1264–1270. 10.1167/iovs.11-9063 22266524

[15] Xu L, Blonska AM, Pumariega NM, ohrab MA, Hageman GS, Smith RT. Reticular macular disease is associated with multilobular geographic atrophy in age-related macular degeneration. *Retina* 2013;33:1850–1862. 10.1097/IAE.0b013e31828991b2 PMC3784629 23632954

[16] Marsiglia M, Boddu S, Chen CY, Jung JJ, Mrejen S, Gallego-Pinazo R, et al. Correlation between neovascular lesion type and clinical characteristics of nonneovascular fellow eyes in patients with unilateral, neovascular age-related macular degeneration. *Retina* 2015;35:966–974. 10.1097/IAE.0000000000000460 25627089

[17] Buitendijk GHS, Hooghart AJ, Brussee C, de Jong PT, Hofman A, Vingerling JR, et al. Epidemiology of reticular pseudodrusen in age-related macular degeneration: the Rotterdam Study. *Invest Ophthalmol Vis Sci* 2016;57:5593–5601. 10.1167/iovs.15-18816 27768796

[18] Joachim N, Mitchell P, Rochtchina E, Tan AG, Wang JJ. Incidence and progression of reticular drusen in age-related macular degeneration: findings from an older Australian cohort. *Ophthalmology* 2014;121:917–925. 10.1016/j.ophtha.2013.10.043 24332537

[19] Suzuki M, Sato T, Spaide RF. Pseudodrusen subtypes as delineated by multimodal imaging of the fundus. *Am J Ophthalmol* 2014;157:1005–1012. 10.1016/j.ajo.2014.01.025 24503406

[20] Curcio CA, Sloan KR, Kalina RE, Hendrickson AE. Human photoreceptor topography. *J Comp Neurol* 1990;292:497–523. 10.1002/cne.902920402 2324310

[21] Ooto S, Ellabban AA, Ueda-Arakawa N, Oishi A, Tamura H, Yamashiro K, et al. Reduction of retinal sensitivity in eyes with reticular pseudodrusen. *Am J Ophthalmol* 2013;156:1184–1191.e2. 10.1016/j.ajo.2013.06.036 23972310

[22] Querques G, Querques L, Forte R, Massamba N, Coscas F, Souied EH. Choroidal changes associated with reticular pseudodrusen. *Invest Ophthalmol Vis Sci* 2012;53:1258–1263. 10.1167/iovs.11-8907 22222508

[23] Garg A, Oll M, Yzer S, Chang S, Barile GR, Merriam JC, et al. Reticular pseudodrusen in early age-related macular degeneration are associated with choroidal thinning. *Invest Ophthalmol Vis Sci* 2013;54:7075–7081. 10.1167/iovs.13-12474 PMC3813319 24071958

[24] Ueda-Arakawa N, Ooto S, Ellabban AA, Takahashi A, Oishi A, Tamura H, et al. Macular choroidal thickness and volume of eyes with reticular pseudodrusen using swept-source optical coherence tomography. *Am J Ophthalmol* 2014;157:994–1004. 10.1016/j.ajo.2014.01.018 24491418

[25] Corvi F, Souied EH, Capuano V, Costanzo E, Benatti L, Querques L. Choroidal structure in eyes with drusen and reticular pseudodrusen determined by binarisation of optical coherence tomographic images. *Br J Ophthalmol* 2017;101:348–352. 10.1136/bjophthalmol-2016-308548 27190128

[26] Cohen SY, Dubois L, Tadayoni R, Delahaye-Mazza C, Debibie C, Quentel G. Prevalence of reticular pseudodrusen in age-related macular degeneration with newly diagnosed choroidal neovascularisation. *Br J Ophthalmol* 2007;91:354–359. 10.1136/bjo.2006.101022 PMC1857688 16973663

